# On the effects of impulsivity and compulsivity on neural correlates of model-based performance

**DOI:** 10.1038/s41598-024-71692-w

**Published:** 2024-09-10

**Authors:** Kerstin Dück, Raoul Wüllhorst, Rebecca Overmeyer, Tanja Endrass

**Affiliations:** 1 Faculty of Psychology, Chair for Addicition Research, Technische Universität Dresden, 01062 Dresden, Germany; 2 Neuroimaging Center, Technische Universität Dresden, 01062 Dresden, Germany

**Keywords:** Psychology, Human behaviour, Computational neuroscience, Reward, Cognitive neuroscience, Cognitive control

## Abstract

Impaired goal-directed behavior is associated with a range of mental disorders, implicating underlying transdiagnostic factors. While compulsivity has been linked to reduced model-based (MB) control, impulsivity has rarely been studied in the context of reinforcement learning despite its links to reward processing and cognitive control. This study investigated the neural mechanisms underlying MB control and the influence of impulsivity and compulsivity, using EEG data from 238 individuals during a two-step decision making task. Single-trial analyses revealed a modulation of the feedback-related negativity (FRN), where amplitudes were higher after common transitions and positive reward prediction error (RPE), indicating a valence effect. Meanwhile, enhanced P3 amplitudes after rare transitions and both positive and negative RPE possibly reflect surprise. In a second step, we regressed the mean *b* values of the effect of RPE on the EEG signals onto self-reported impulsivity and compulsivity and behavioral MB control (*w*). The effect of RPE on FRN-related activity was mainly associated with higher *w* scores, linking the FRN to MB control. Crucially, the modulation of the P3 by RPE was negatively associated with compulsivity, pointing to a deficient mental model in highly compulsive individuals.

## Introduction

Learning and decision-making are assumed to be influenced by two reinforcement learning mechanisms: model-free (MF) and model-based (MB) learning. MF learning relies on past action-reward experiences, leading to reward prediction errors (RPE) when expected outcomes do not occur. RPEs alter the value of a choice option and subsequently influence its selection probability^1–4^. MF control facilitates performance with little cognitive effort, but adjustments are slow and it is linked to habitual and inflexible behavior^5^. In contrast, MB learning employs a mental model of a given task, mapping associations of actions and potential outcomes^1–4^. Despite being more computationally demanding, MB learning facilitates goal-directed behavior in complex environments^6,7^. Both systems operate in parallel, and the balance between them, computationally quantified by the weighting parameter *w*, varies with inter-individual differences and contextual factors^1,2,4^.

Shifts towards MF learning can lead to suboptimal choices, and an imbalance between MF and MB control has been observed in various mental disorders, e.g. substance use disorder^8,9^, schizophrenia^10^, gambling disorder^11^, anorexia nervosa^12^, or obsessive–compulsive disorder^13,14^. This range of disorders, along with changes in MF and MB control in non-clinical samples^15^, suggests that dysfunction in goal-directed behavior may reflect common underlying mechanisms.

Impulsivity and compulsivity are strong candidates for transdiagnostic factors: Impulsivity, characterized by a tendency toward rapid, unplanned actions^16^, is associated with reward seeking and behaviors carrying the risk of harm, such as gambling or aggressive actions^17–19^. Heightened impulsivity is also linked to SUD^20^ and other disorders characterized by altered reinforcement learning^21–23^. The associations of impulsivity with cognitive control^24–26^ and decision making^27–29^ have been studied extensively. Although research directly linking reinforcement learning and impulsivity is limited, existing studies suggest associated dysfunctions^30,31^. Conversely, compulsivity, involving the tendency to repeat actions despite adverse consequences^32^, is linked to deficits in MB control in the general population^33^ and various mental disorders within the compulsivity spectrum^34^. Impulsivity and compulsivity have been found to overlap significantly in both neurocircuitry^35^ as well as associations with psychopathology, particularly disorders implicated with altered MB control^36,37^. Although these traits have been found to interact at both the psychophysiological^38^ and symptom levels^39,40^, prior studies have typically examined their effects on MB control independently, without considering their possible interplay.

**Fig. 1:**
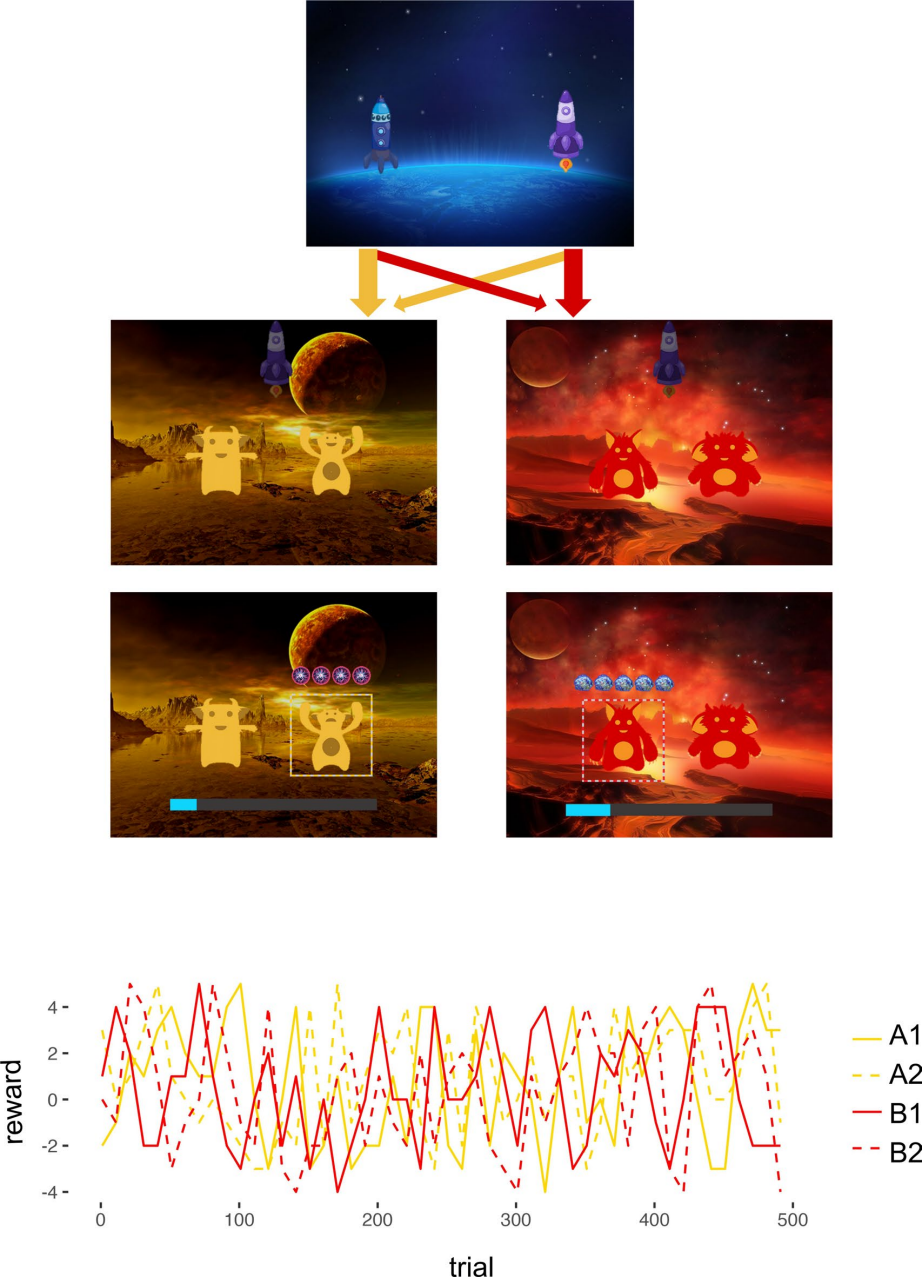
Two-step task. Top: Schematic illustration of the task First row: In stage one, participants chose one of two stimuli (spaceships), which led them to one of two second stages (planets) with a probability of 80% (common; depicted on the right) or 20% (rare; depicted on the left), respectively. Second row: In stage two, participants again chose between two stage-specific stimuli (aliens). Third row: The chosen stimulus was then marked. Each second-stage choice resulted in a gain or loss of a varying number of points, which was displayed above the stimulus, in addition to their point total, indicated as a bar at the bottom. Comparisons between the expected and actually obtained reward (points) at each trial then resulted in positive, negative or no/neutral reward prediction errors. Bottom: Rewards for each stimulus (1 or 2) on each second stage (A or B) changed over time.

To address this gap, research considering both dimensions within reinforcement learning paradigms is highly warranted to delineate their individual effects and possible interactions. In this study, we employed a sequential decision-making task (two-step task), which is a task designed to investigate MB learning. In its original version^2^, participants are given the choice of two stimuli in a first stage, which then leads them to one of two second stages, each with another distinct stimulus pair to choose between. The transition between the first and second stage is probabilistic. Each choice is linked to one second stage with a high probability (common transition) and to the other second stage with a low probability (rare transition). In the second stage, participants choose between two stimuli resulting in monetary reward or loss, with the outcome changing over time (Fig. 1). MF and MB learners can be differentiated by the extent they consider the transition structure of the task in their first-stage decisions. Since MF learners are assumed to base the perceived value of actions on previous reward, choices in the first stage are likely to be repeated if participants were subsequently rewarded in the second stage, regardless of transition type. In contrast, MB control relies on action-outcome associations, where learners consider both the obtained reward and the transition type from the previous trials. For example, if a reward follows a rare transition, MB learners are more likely switch rather than stay, as they predict a higher likelihood of reward by choosing the alternative first-stage option.

We examined the neural mechanisms of reinforcement learning during the two-step task and their relation to impulsivity and compulsivity, using electroencephalography (EEG). The feedback-related negativity (FRN) occurs fronto-centrally 200–350 ms after feedback presentation and is associated with reward prediction errors^41–43^, which MF control is based on. Given that impulsive individuals tend to prioritize immediate rewards^44^ and show more MF behavior^30^, a close relationship to the FRN appears likely. The centroparietal P3, a positive-going component starting around 300 ms after stimulus presentation, shows associations with the probability and relevance of stimuli for building and updating representations of the environment^45^. In sequential decision making paradigms, the P3 should thus be linked to the representation of the task structure. P3 amplitude has been found to vary with transition structure^46,47^ and RPE^47^, both integral for a mental model of the task. Task representation deficits reflected in the EEG have been linked to reduced MB planning in individuals with higher compulsivity^48^.

As feedback processing is central for both MF and MB control in reinforcement learning, altered RPE signaling is expected to underlie dysfunctions in decision making. We thus investigated how RPE signals (FRN, P3) in a two-step task were modulated by both impulsivity and compulsivity. Using single-trial analyses, we examined the coupling between RPE and brain activity focusing on the time-windows and locations associated with the FRN and P3. Considering the associations between RPE, FRN, and impulsivity, we hypothesized that the RPE modulation of the FRN would increase with higher impulsivity. Meanwhile, because compulsivity is considered to impede task representation, we expected a diminished effect of RPE on the P3 in individuals with high compulsivity. Lastly, we explored potential interaction effects of impulsivity and compulsivity.

## Results

### Behavior

We employed a logistic mixed-effect model to investigate whether participants’ first-stage choices showed characteristics of model-free and model-based behavior, and to assess the impact of impulsivity and compulsivity on these tendencies. Characteristics of the previous trial (reward and transition type) and participants’ BIS-11 and OCI-R scores were regressed onto stay probability. As shown in Table 1, participants showed significant effects for reward (β = 1.27, *p* =  < 0.001) and reward*transition interaction (β = 2.01, *p* =  < 0.001), indicating that both MF and MB learning determined behavior. Neither the BIS-11 nor the OCI-R showed significant effects and there was no effect of gender.

**Table 1:** Fixed effects of the generalized linear mixed-effects model for stay probability in stage 1.

	*β*	*SE*	*t*	*p*
(Intercept)	3.10	.12	30.4	< .001
Reward	1.27*	.02	15.8	< .001
Transition	1.09*	.02	6.14	< .001
BIS	1.03	.03	.83	.404
OCI	1.05	.03	1.71	.081
Gender	.95	.04	− 1.03	.305
Reward*transition	2.01*	.04	34.0	< .001
Reward*BIS	1.02	.02	1.62	.105
Transition*BIS	1.02	.01	1.46	.145
Reward*OCI	1.00	.02	− .02	.987
Transition*OCI	.98	.01	− 1.17	.245
BIS*OCI	1.03	.03	.92	.357
Reward*transition*BIS	.98	.02	− .88	.379
Reward*transition*OCI	1.01	.02	.28	.779
Reward*BIS*OCI	1.00	.02	.30	.761
Transition*BIS*OCI	.98	.01	− 1.80	.072
Reward*transition*BIS*OCI	.99	.02	− .37	.711

Reward = reward in the previous trial (yes: 1|no: − 1.) Transition = transition type of the previous trial (common: 1|rare: − 1). BIS = z-scored sum score Barratt Impulsiveness Scale. OCI = z-scored sum score Obsessive Compulsive Inventory Revised *β* = exponentiated *β* weight after logistic regression.

*p < .05.

Further, we explored the association of RT differences (rare minus common) as a marker for MB learning with impulsivity and compulsivity. Regression analysis revealed neither main nor interaction effects (see supplementary table S2).

### EEG data

#### First-level results: Task effects

**Fig. 2:**
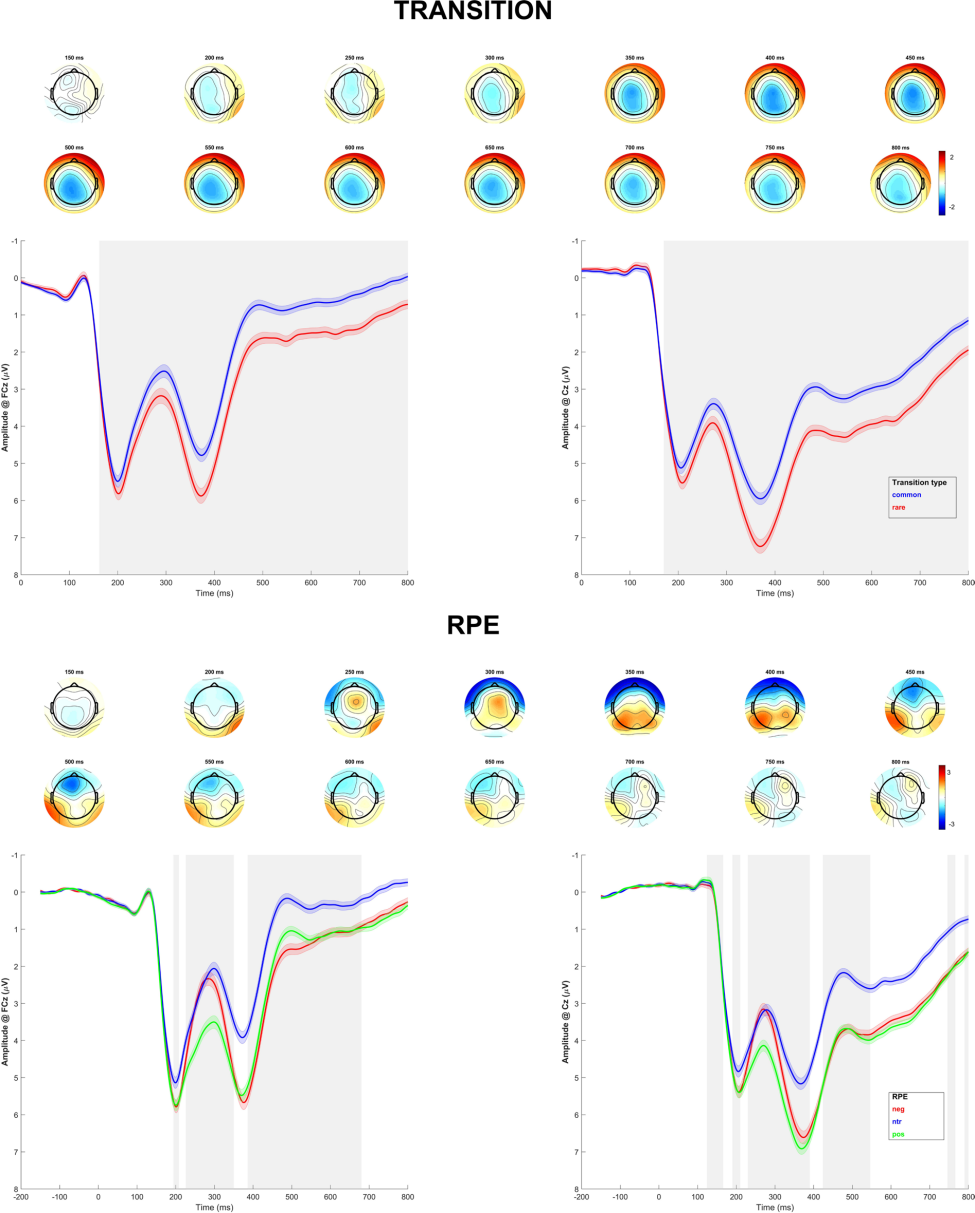
First-level regression of transition type and RPE. First–second and fourth-fifth row: Topography of the *b* values for the first-level effect (150–800 ms; top: transition, bottom: RPE). Third and sixth row: EEG time course at FCz (left) and Cz (right), split into common and rare transitions (top) or RPE terciles for visualization (bottom). Shading indicates SEM. EEG activity is locked to second-stage feedback presentation. Gray shading behind EEG activity indicates significance of regression weights after FDR-correction. RPE = reward prediction error. Neg = negative RPE, ntr = neutral/no RPE, pos = positive RPE. Mean RPE in first tercile (negative): *m* = − 0.260, SD = 0.018, mean RPE in second tercile (neutral): *m* = − 0.005, *SD* = 0.023, mean RPE in third tercile (positive): *m* = 0.290, *SD* = 0.030.

**Fig. 3:**
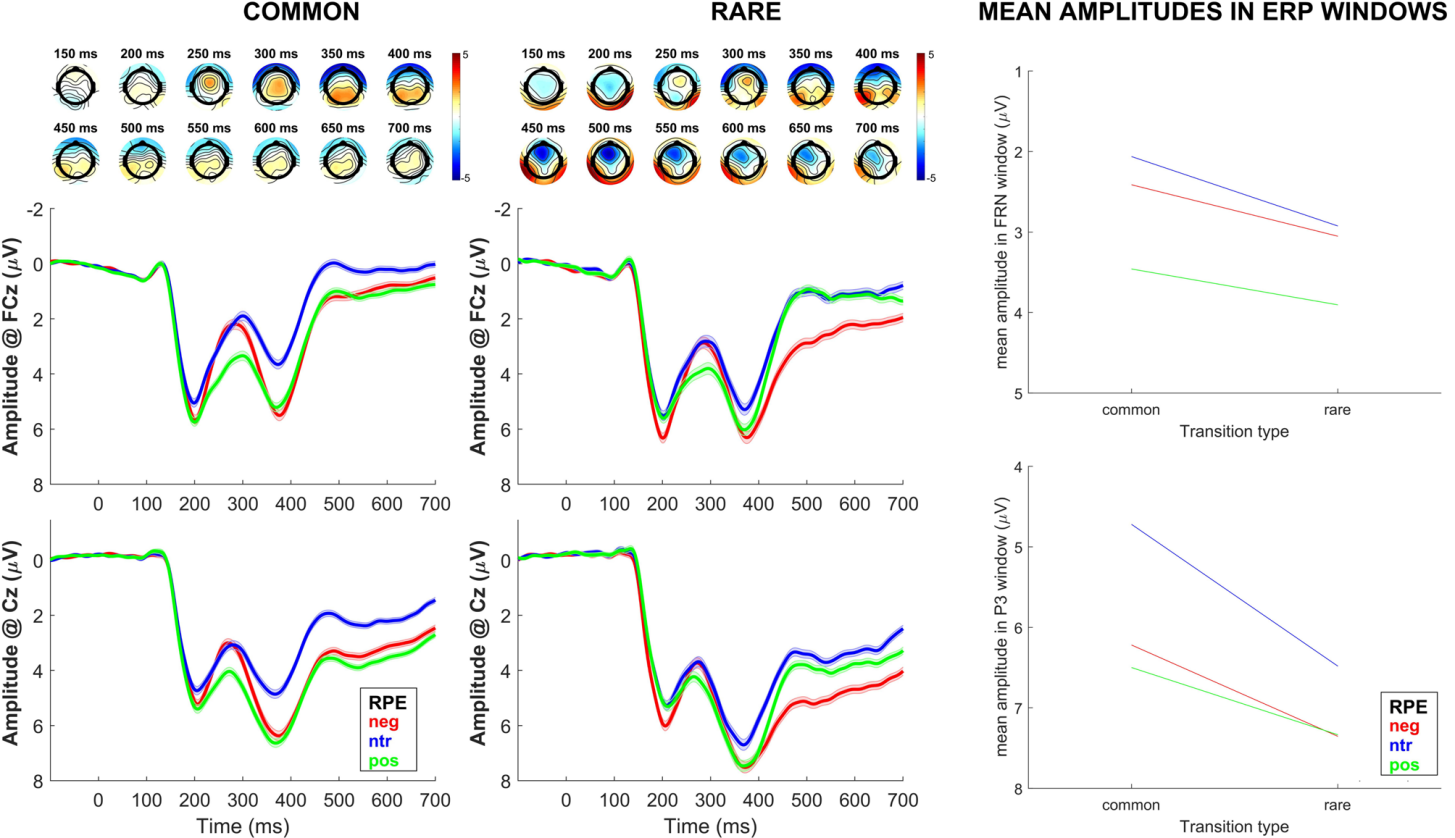
First-level regression of RPE split into trial types. For left and middle column: Data for common and rare transitions. First and second row: Topography of the *b* values for the RPE effect (150–800 ms). Third and fourth row: EEG time course at FCz and Cz, split into RPE terciles for visualization. Shading indicates SEM. EEG activity is locked to second-stage feedback presentation. For right column: Mean EEG amplitudes in FRN time-window at FCz (top) and P3 time-window at Cz (bottom). Lineplots show interaction of transition type and RPE. RPE = reward prediction error. Neg = negative RPE, ntr = neutral/no RPE, pos = positive RPE. Mean RPE in first tercile (negative): *m* = − 0.260, SD = 0.018, mean RPE in second tercile (neutral): *m* = − 0.005, *SD* = 0.023, mean RPE in third tercile (positive): *m* = 0.290, *SD* = 0.030.

The effects of transition type and RPE on second-stage EEG data were analyzed using single trial regression to obtain a regression weight time-course for all electrodes (Fig. 2). For transition type, we observed a significant negative effect starting around 180 ms after feedback onset, indicating more positive EEG signals for rare compared to common trials. Thus, the FRN at FCz was more negative for common trials (*β*_mean_ = − 0.62, *p* =  < 0.001), while the P3 was more positive for rare trials (*β*_mean_ = − 1.25, *p* =  < 0.001 at Cz). Single-trial regression additionally revealed a significant effect of RPE during the FRN time window (*β*_mean_ = 1.63, *p* =  < 0.001 at FCz) characterized by lower (less negative) amplitudes for positive RPEs as compared to negative RPEs. We also observed a significant RPE effect for P3 (*β*_max_ = 0.55, *p* =  < 0.001 at Cz), indicating that amplitudes were higher (more positive) for both positive and negative RPE. There also were significant transition*RPE interaction effects in the FRN (*β*_mean_ = 0.933, *p* =  < 0.001 at FCz) and P3 (*β*_mean_ = 0.88 at 356 ms, *p* =  < 0.001 at Cz) time windows (see supplementary figure S1). As depicted in Fig. 3, the RPE effects were stronger for common trials at FCz and Cz.

#### Second-level results: Impulsivity and compulsivity effects

**Fig. 4:**
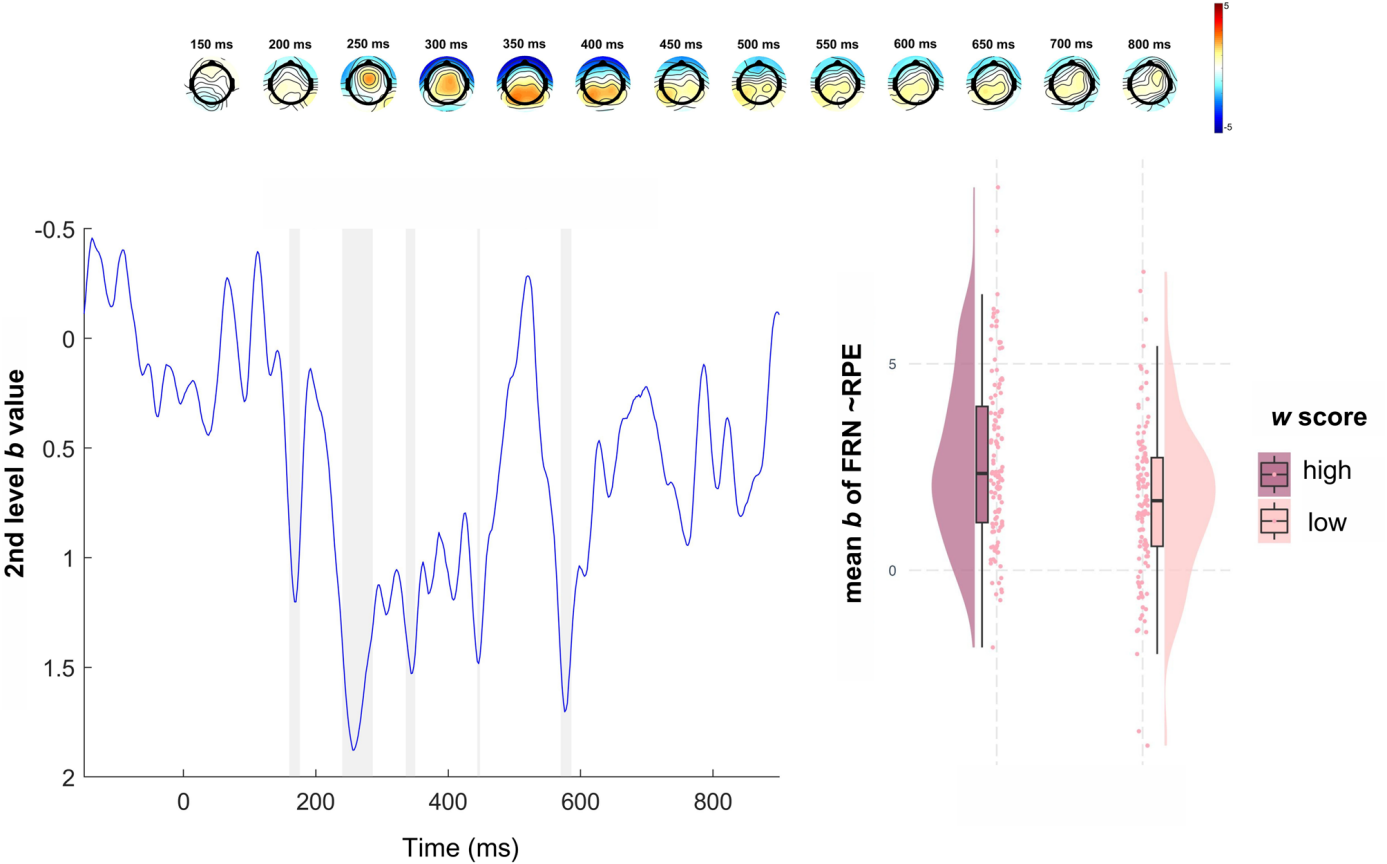
Second-level regression of *w*. First row: Topography of the *b* values for the 2nd level regressor *w* (150–700 ms). Second row, left: Time course of 2nd level regressor *w* at FCz. Gray shading indicates significance of regression weights after FDR-correction. Second row, right: Mean *b* values of RPE effect in FRN time-window at FCz after common transitions for groups of high (left) and low (right) *w* score. Raincloud plot shows data distribution, jittered raw data and boxplot.

**Fig. 5:**
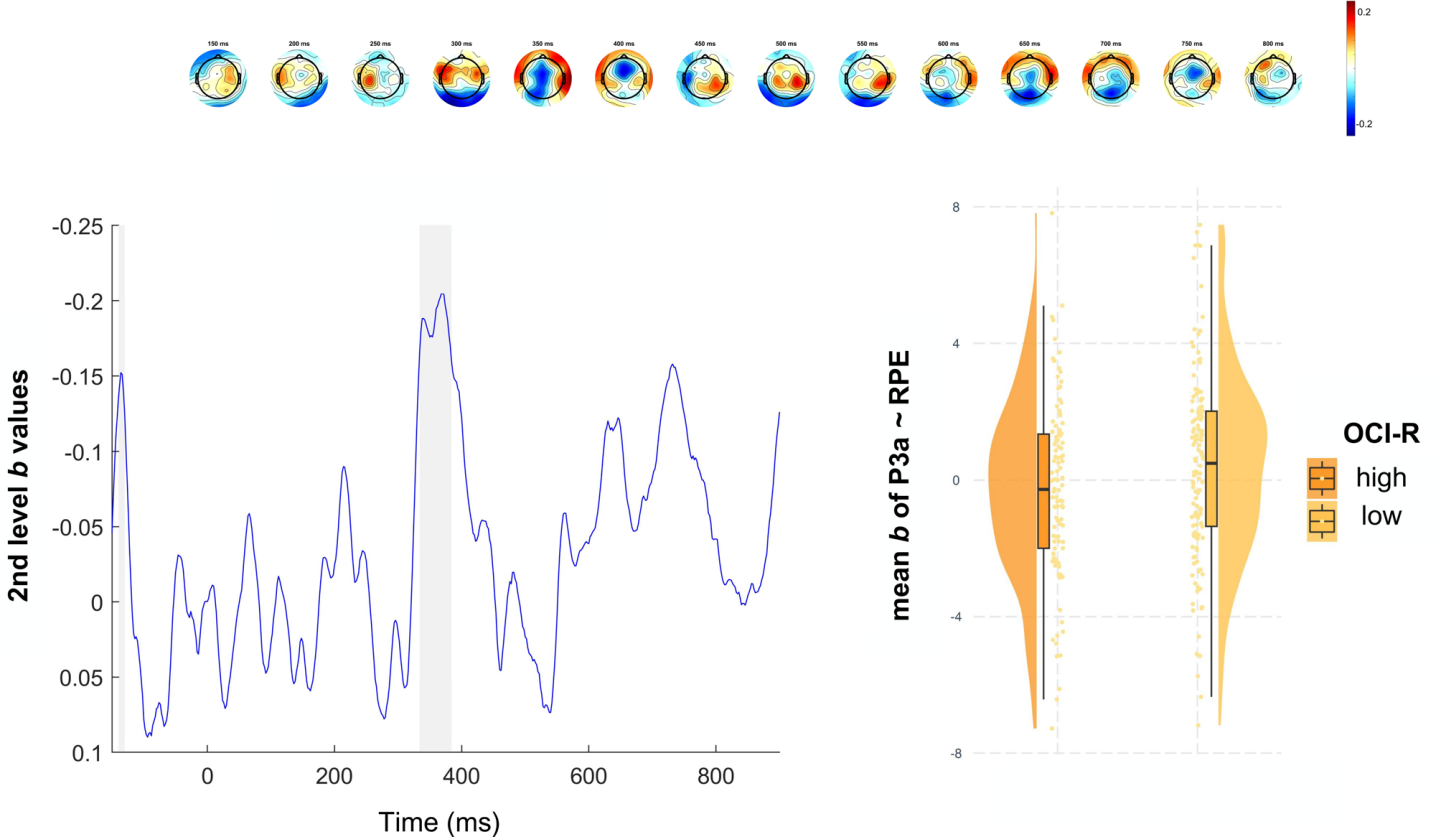
Second-level regression of OCI-R. First row: Topography of the *b* values for the 2nd level regressor OCI-R (150–700 ms). Second row, left: Time course of 2nd level regressor OCI-R at Cz. Gray shading indicates significance of regression weights after FDR-correction. Second row, right: Mean *b* values of RPE effect in P3 time-window at Cz after rare transitions for groups of high (left) and low (right) OCI-R score. Raincloud plot shows data distribution, jittered raw data and boxplot.

**Fig. 6:**
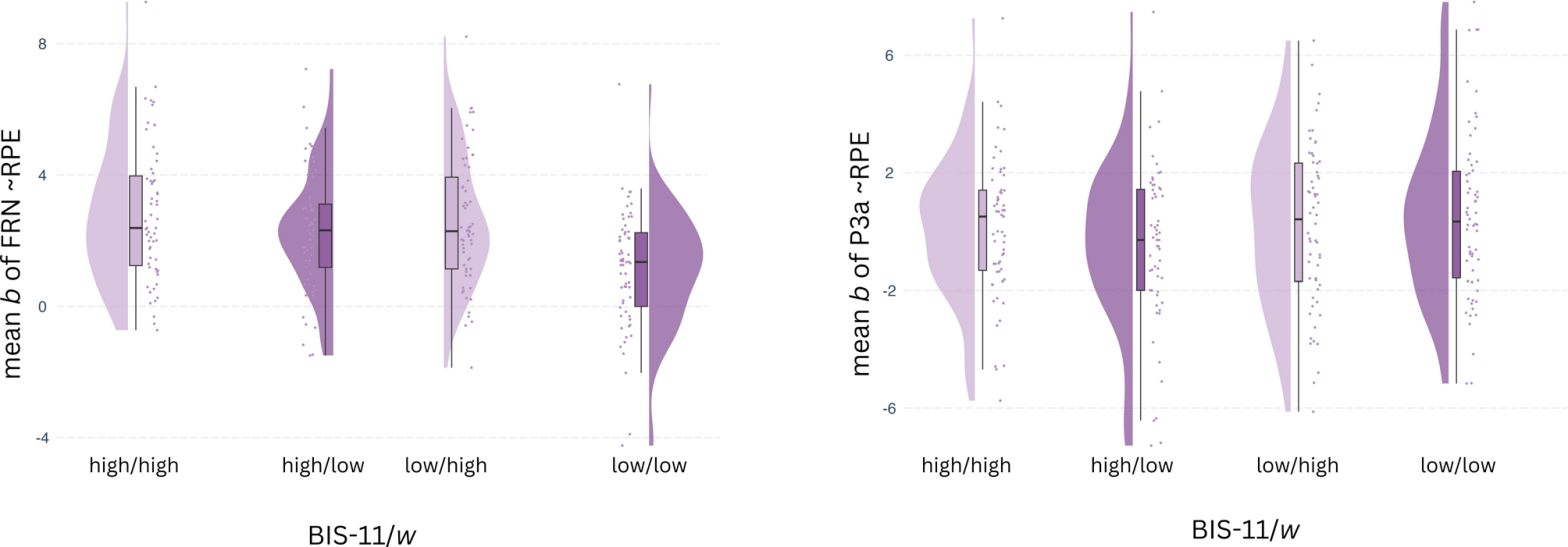
Regression of RPE in combined (median split) groups of high and low impulsivity and *w.* Left: Mean *b* values of RPE effect during second-stage feedback presentation in FRN time-window at FCz. Raincloud plot shows data distribution, jittered raw data and boxplot. Right: Mean *b* values of RPE effect in P3 time-window at Cz. Raincloud plot shows data distribution, jittered raw data and boxplot.

We examined how self-reported impulsivity and compulsivity, as well as *w*, modulated the first-level effect of RPE on the EEG signal during the FRN and P3 in common and rare trials (Table 2). The RPE effect in the FRN window was significantly positively associated with *w* scores (common trials: *β* = 0.31, *p* = 0.023; rare trials: *β* = 0.33, *p* = 0.048), indicating larger RPE-related amplitude modulations in individuals with higher *w* scores (see Fig. 4). We also found a trend level effect of BIS-11 scores on FRN-related activity (*β* = 0.23, *p* = 0.073) and an interaction of BIS-11 and *w* scores (*β* = − 0.26, *p* = 0.029) after common transitions. Furthermore, the RPE effect on P3-related activity (see Fig. 5) on rare trials was negatively associated with OCI-R scores (*β* = − 0.45, *p* = 0.010), indicating reduced RPE-related amplitude modulations in individuals with higher compulsivity. Additionally, we observed a significant interaction of BIS-11 and *w* scores (*β* = 0.39, *p* = 0.015) for the RPE effect on P3-related activity. To follow up on the interactions of BIS-11 and *w,* we computed regressions of each predictor (BIS-11 or *w*) in median split (high vs. low) groups of the other (Fig. 6 and supplementary table S3). For the FRN, both BIS-11 and *w* showed positive associations with the RPE effect (*β*_BIS-11_ = 0.40 and *β *_*w*_ = 0.49) when the other predictor was held low, which were dampened with high BIS-11 (*β *_*w*_ = 0.17) and high *w* (*β*_BIS-11_ = − 0.03) scores. Regarding the effect of RPE on P3-related activity, we found a positive link with *w* in the high impulsivity group (*β*_*w*_ = 0.34), whereas the association in the low impulsivity group was reduced (*β*_*w*_ = − 0.09). BIS-11 showed a negative association in the low *w* group (*β*_BIS-11_ = − 0.43) which was reversed with high *w* (*β*_BIS-11_ = 0.22).

**Table 2:** Robust linear regression results for common and rare trials.

		Common				Rare			
	Regressor	*β*	*SE*	*t*	*p*	*β*	*SE*	*t*	*p*
FRN	(Intercept)	2.09	.13	16.31	< .001	1.16	.16	7.38	< .001
	BIS	.23	.13	1.80	.073	.06	.16	.39	.702
	OCI	− .12	.13	− .96	.331	.03	.16	.22	.831
	*w*	.31*	**.14**	**2.25**	**.023**	.33*	**.17**	**1.96**	**.048**
	BIS*OCI	.11	.13	.83	.403	− .08	.16	− .50	.620
	BIS*w	− .26*	**.13**	− **2.17**	**.029**	.01	.16	.07	.943
	OCI*w	.23	.12	1.55	.116	.23	.15	1.24	.210
	BIS*OCI*w	− .11	.16	− .71	.466	.02	.19	.11	.910
P3	(Intercept)	1.01	.14	7.02	< .001	.06	.17	.37	.713
	BIS	.17	.15	1.14	.254	− .10	.17	− .60	.547
	OCI	− .12	.14	− .83	.404	− .45*	**.17**	− **2.61**	**.010**
	*w*	.27	.15	1.74	.081	.23	.18	1.26	.203
	BIS*OCI	.06	.14	.41	.682	.04	.17	.25	.802
	BIS*w	− .02	.13	− .13	.899	.39*	**.16**	**2.43**	**.015**
	OCI*w	− .06	.17	− .34	.735	.10	.20	.48	.624
	BIS*OCI*w	.03	.18	.16	.874	.13	.21	.62	.525

Common = trials with common transition. Rare = trials with rare transition. FRN = mean *b* values of RPE effect in the time-window for feedback-related negativity at FCz. P3 = mean *b* values of RPE effect in the time-window for P3 at Cz. BIS = z-scored sum score Barratt Impulsiveness Scale-11. OCI = z-scored sum score Obsessive Compulsive Inventory-Revised. *W* = z-scored weighting parameter.

*p < .05, values marked in boldface.

When we compared regression coefficients against each other using z-tests, only the effects of *w* on the FRN in varying levels of BIS-11 differed significantly (see supplementary table S3).

## Discussion

In this study, we investigated the association of EEG correlates of outcome processing during a two-step decision-making task with interindividual differences in impulsivity, compulsivity and MB control (*w*). Single-trial regression revealed that FRN-related activity exhibited higher amplitudes following common transitions and reduced amplitudes after positive RPE. In contrast, the P3 showed enhanced amplitudes after rare transitions and in response to both positive and negative RPE. The effect of RPE on the FRN was positively associated with *w* scores, while the effect of the RPE on P3 activity showed a negative correlation with OCI-R scores following rare transitions. Furthermore, both the FRN and P3 effects were related to the BIS-11*w interaction.

Enhanced FRN amplitudes following common transitions, compared to rare transitions, point to a relationship between the FRN and MB control: Based on a mental model of the environment, it would be favorable to discern between transition types and rely more heavily on common trials, as they give more information on accumulating reward and overall task output. In contrast, the P3 was enhanced on rare trials, which is in line with the general assumption that the P3 reflects stimulus salience and probability^49,50^ as well as context updating^51^. Signed RPE further revealed distinct effects on the FRN and P3. Whereas the FRN was less pronounced with positive RPE, the P3 was larger for both positive and negative RPE. Larger FRN amplitudes for neutral and negative RPEs, compared to positive RPEs, suggest that this modulation is driven by positive prediction errors. This supports the view that the FRN reflects feedback valence^17,52^, rather than RPE magnitude or „unsigned “ RPE, as some researchers have suggested^43,53,54^. However, our findings also align with the concept of the reward positivity (RewP). The RewP is assumed to capture an amplitude modulation attributed to rewards instead of negative or neutral feedback^55,56^. While it is easily visible as a difference wave, the RewP has been suggested to be masked by the N2 component^57,58^, potentially creating the negative deflection observed following negative feedback and also apparent in our data, which is positively modulated only by reward feedback. In the following discussion, we will thus consider both the FRN and the RewP. Additionally, higher P3 amplitudes after positive and negative RPEs suggest a role of surprise, consistent with the P3’s association with mental task representation^48^. Lastly, the transition*RPE interaction revealed stronger RPE effects following common transitions relative to rare transitions for all investigated ERPs. As described above, tracking RPE after common transitions is more informative for overall reward, suggesting MB control.

In second-level analyses, P3-related activity displayed a modulation by compulsivity: individuals with high OCI-R scores showed a reduced influence of RPE on the P3. Notably, this effect appeared only after rare transitions, pointing to a reduced use of RPEs from these trials to update choice values. As this process relies on integrating transition information and RPE and is based on a mental task representation, it may indicate impaired MB control. Supporting evidence for an association of high compulsivity with deficient mental models comes from several neuroimaging studies in compulsivity-related disorders^59^: For example, smokers processed prediction errors from fictive outcomes, yet did not adapt their behavior accordingly^60^, while alcohol-dependent individuals showed reduced activation related to the updating of different choice options^61^.

Our findings align with those of Seow et al.^48^, who observed that P3, alpha power and RT after second-stage stimulus presentation were all sensitive to transition type, although only the latter two were associated with MB control and compulsivity. The authors propose that reduced orienting processes (reflected in RT and alpha power) indicate a deficient representation of transition structure, pointing to an impaired mental model. This relationship between behavioral and neural responses to transition type and mental task representation appears more visible in stimulus-locked data. Meanwhile, our data suggests neural deficits in the mental model after feedback. It is possible that compulsivity affects MB control at both levels, with stimulus- and feedback-locked analyses offering complementary insights into these deficits.

MB control was also relevant for FRN/RewP-related activity, as we found that higher *w* scores modulated the effect of RPE. This is consistent with the notion that *w* reflects a tendency toward MB control, because RPE is not merely based on feedback valence, but instead requires a mental task representation to form predictions. According to this mental model, tracking RPE in common trials is more crucial because it impacts the overall points obtained. Thus, the FRN/RewP appears as both a reflection of the integration of RPE into MB learning as well as reward processing related to MF learning. This overlap is consistent with neuroimaging finding, where MF and MB signals are not entirely distinct; e.g. with the ventral striatum, a region associated with reward processing^17^, has been shown to reflect both types of control^2^.

BIS-11 scores showed a trend-level effect on RPE modulation, possibly because impulsivity is associated with reward processing^17,18,63^ and might influence RPE to some extent, it primarily impacts MF rather than MB learning, thus limiting its effect on RPE modulation. However, the regression effects of BIS-11 and *w* were qualified by an interaction on common trials, which was followed up by regression analyses of each predictor in median split (high vs. low) groups of BIS-11 or *w* scores, respectively. Results showed that the effect of *w* differed significantly between levels of BIS-11, with a positive relationship between *w* and the FRN/RewP only when impulsivity was low. Impulsivity has been associated with reduced goal-directed control^64,65^, whereas high *w*, as an indicator of model-basedness, is related to reduced MF learning. The interaction effect suggests that impulsivity is more linked to MF control and that the FRN/RewP reflects not only MF, but also MB control, consistent with findings that the RewP signals the integration of complex action sequences^66^. Post-hoc analyses also revealed changes in the effects of BIS-11 on the FRN/RewP and both predictors on the P3 that further depict the BIS-11*w interaction. Although differences in regression scores from median split groups did not reach significance, the interaction effects point to an interplay between impulsivity and markers of goal-directed control, requiring further research to disentangle their specific effects.

Behavioral analyses focused on participants’ tendency to repeat a first-stage decision. The linear mixed models revealed effects of the previous trial’s outcome and transition (reward and reward*transition interaction), pointing to the well-established mixture of MF and MB control^46,48,67^. Yet, unlike some previous studies, we did not observe interaction effects with BIS-11 and OCI-R scores. For example, Deserno et al.^30^, reported the reward effects on stay probabilities to vary with impulsivity, but their study compared extreme groups of impulsivity within a smaller, less homogeneous sample, which may have increased effects. Raio et al.^31^ found impulsivity scores to affect choice behavior only in the second stage of a two-step task, where participants can be influenced by previous reward, but MF and MB control are no longer differentiated. Thus, these results on decision-making deficits may not apply to our analyses. Seow et al.^48^ found that individuals with higher compulsivity scores had a reduced prolongation of reaction times after rare vs. common transitions , which was interpreted as impaired mental task representation. We could not replicate this result in our study. Possibly, effects of impulsivity and compulsivity on outcome processing on the neural level do not translate directly into participants’ first-stage choice behavior. This might be due to compensatory mechanisms, e.g. high cognitive effort. Outside of a controlled laboratory setting, where various internal and external factors challenge resources, individuals might find it more difficult to maintain MB control.

One limitation of our study is the potential impact of gender. Literature indicates that impulsivity scores vary by gender^68^ and there is evidence that gender moderates the association of trait impulsivity and risk behavior^69,70^. In reinforcement learning, gender differences have been found behaviorally^71^ and in the relationship between stress and cortico-striatal brain function^72^, suggesting possible confounding effects. However, since our study did not find a gender effect at the behavioral level, we did not include gender in our EEG analyses to maintain models parsimonious. Future studies are warranted to address this gap in reinforcement learning studies.

In conclusion, we found feedback processing in the EEG to be modulated by interindividual differences. As expected, RPE had less influence on the P3 in highly compulsive individuals, which we interpreted as a deficient mental model. However, further research is needed on the nature of the mental model deficits, i.e. if the model itself is not built or not applied properly. Although impulsivity is often connected to poor decision-making outside of the laboratory, the association in our task was only weak. More research to bridge this gap to “real-life” deficits is thus highly warranted. Still, based on our large community sample, our results may help explain impaired decision-making as seen in reduced MB control in a specific task as well as the everyday behavior that characterizes compulsivity in both clinical and healthy populations.

## Methods

### Participants

Two-hundred fifty-three participants from the general Dresden area participated in the study. Inclusion criteria were age 18–45 years, native German speakers, and normal or corrected-to-normal vision. Participants were excluded if they reported a history of neurological disorder or head trauma; lifetime diagnosis of bipolar disorder, borderline personality disorder, psychotic episodes, or severe alcohol use disorder; acute eating disorder or severe episode of major depression; psychotropic medication within the last three months; lifetime use of illicit substances more than twice a year or lifetime use of cannabis more than twice a month. We excluded participants from analysis due to poor task compliance (*N* = 11), technical EEG recording errors (*N* = 3), and retrospective identification of exclusion criteria (cannabis use, *N* = 1). Thus, the final sample consisted of 238 participants (50% female; age *M* = 25.04, *SD* = 4.84; education level: 95% high school or higher).

The project has been approved by the ethics committee at the University Hospital Carl Gustav Carus, at TU Dresden (EK 372092017) and was conducted in accordance with the ethical guidelines of the Declaration of Helsinki. All participants gave informed consent and received financial compensation (80–100 €) or course credit for participation. The study is part of a larger research project which assessed different cognitive control functions in relation to impulsivity and compulsivity (https://osf.io/ywnze/).

### Procedure and measures

The two-step task was completed as part of an EEG session in the lab. Additionally, participants completed other EEG tasks as well as a neuropsychological test battery at a first lab appointment, and ecological momentary assessment, which will not be reported here.

#### Two-step task

Participants performed a modified two-step task^2,7^, consisting of two subsequent decision making stages. First, they were asked to choose one of two first-stage stimuli (cartoon drawings of spaceships), which would then lead them to one of two possible second stages (planets). Each first-stage stimulus was associated with a second stage with a transition probability of 80% (common transition) or 20% (rare transition), respectively (see Fig. 1). In the second stage, participants were presented with a set of two stimuli (aliens) specific to the particular stage, and again instructed to choose one, resulting in a number of points added to or subtracted from their total count. The reward probability at stage two followed a random walk with reflective bounds at + 5 and − 4 points. Participants were told that choosing one alien meant asking it to dig for space treasure. The aliens then presented them with up to 5 pieces of space treasure (adding points) or up to 4 pieces of “anti-matter” (subtracting points), or nothing (no points). The goal was to collect as much treasure, i.e. points, as possible, which would be transformed into a bonus of up to 5 € at the end of the task.

The task was composed of 500 trials split into four blocks. First and second-stage stimuli remained on screen until the response (left or right index fingers; max. response window: 2000 ms), the selected option was then marked for 500–800 ms. After the second-stage response, the outcome was displayed for 1000 ms as the respective number of icons for treasure or anti-matter, together with a bar indicating the subject’s total point count. A black screen was shown for 300–800 ms between trials.

Participants were instructed that each spaceship had a fixed preference for one planet and that the outcome of each alien would change over time. They familiarized themselves with the transition and reward structure of the task before completing a block of 25 of practice trials.

#### Personality scales

##### Impulsivity

We used a German translation of the 11th version of the Barratt Impulsiveness Scale (BIS-11)^73^. In 30 self-report items, the questionnaire tests for attentional, motor, and non-planning impulsiveness and yields a sum score with good internal consistency (α = 0.83)^74^.

##### Compulsivity

Compulsivity was operationalized using the Obsessive–Compulsive Inventory-Revised (OCI-R)^75,76^. The self-report questionnaire measures obsessive–compulsive symptom severity (washing, checking, doubting, ordering, obsessing [i.e., having obsessional thoughts], hoarding, and mental neutralizing). We again used the sum score, which has shown good internal consistency^75^. Both BIS-11 and OCI-R scores were z-standardized for further analyses.

### Data acquisition and analysis

Data was analyzed with MATLAB R2021a^77^ and the EEGlab toolbox (version 14.1.2b)^78^ using the high performance computing system (HPC) at the TU Dresden. Further regression analyses were performed with R^79^.

#### Behavioral data and computational modelling

##### First-stage choice data

Participants were excluded from all analyses if they showed random choice behavior (*N* = 11, see sample description), i.e., if the probability to repeat their last first-stage choice (stay probability) was not positively associated with last trial’s reward or reward*transition interaction in a logistic regression model.

Behavioral data was further analyzed using the R package lme4^80^. We tested to what extent the tendency of each participant to repeat their last first-stage choice (stay: 1 | switch: 0) was explained by the transition type (common: 1 | rare: − 1) and reward (yes: 1 | no: − 1) of the previous trial via a logistic mixed-effect model. MF learning, being solely reward-driven, is signified by the tendency to repeat choices after a win and to switch after a loss. In contrast, MB behavior takes the transition structure into account, signified by the tendency to repeat the response after a win if the transition was common, but to switch after a win if the transition was rare. Thus, individual β weights for reward provided an estimate for MF behavior, whereas the interaction between reward and transition type indicated model-based behavior.

It was of particular interest how these terms were associated with BIS-11 and OCI-R scores, to examine how impulsivity and compulsivity affected behavior. Individual intercepts and regression weights for transition, reward, and the transition*reward interaction were included as random effects to allow for variance across participants. In R syntax, the model was: *stay* ~ transition * reward * BIS-11 * OCI-R + (transition * reward | subject).

Based on findings from Seow et al.^48^, we also explored the reaction time (RT) difference for common and rare trials. Surprise after rare transitions, causing an orienting process indicated in longer RT, is based on a mental representation of the transition structure. It can thus be seen as a marker for MB learning. The relationship between RT difference (median RT in rare—median RT in common trials), impulsivity and compulsivity was examined in robust regression (RT_delta_ ~ BIS-11*OCI-R).

##### Computational modelling

Following Kool et al.^7^, the task involved two stages with three possible states s (stage 1: s_A_; stage 2: s_B_ or s_C_), and two possible actions a (a_A_ and a_B_). All models learn to maximize the value Q (s, a). At a given trial *t*, states are denoted as s_1,*t*_ (always s_A_) and s_2,*t*_ (s_B_ or s_C_), actions as a_1,*t*_ and a_2,*t*_, and rewards as r_1,*t*_ (always equal to zero) and r_2,*t*_.

*Model-free.* MF agents solve the task according to the SARSA(λ) temporal difference learning algorithm^81^, such that at each stage *i* and trial *t*

$${Q}_{MF}\left(s,a\right)= {Q}_{MF}\left(s,a\right)+ \alpha {\delta }_{i,t}{e}_{i,t}\left(s,a\right).$$

Here, α denotes the free learning rate parameter (indicating how fast values are updated), δ_*i,t*_ denotes the RPE, and e_*i,t*_(s, a) denotes the free eligibility trace parameter.

As r_1,*t*_ is always equal to zero, the first-stage RPE depends on the second stage action:

$${\delta }_{1,t}= {Q}_{MF}\left({s}_{2,t},{a}_{2,t}\right)- {Q}_{MF}({s}_{1,t},{a}_{1,t})$$

The second-stage RPE depends on r_2,*t*_:

$${\delta }_{2,t}= {r}_{2,t}-{Q}_{MF}\left({s}_{2,t},{a}_{2,t}\right).$$

The eligibility trace equals 0 at the beginning of each trial and is updated before the Q value according to

$${e}_{i,t}\left({s}_{i,t},{a}_{i,t}\right)={e}_{i-1,t}\left({s}_{i,t},{a}_{i,t}\right)+1.$$

First- and second-stage value updates occurred at the second stage. Here, prediction errors of first-stage values were weighted by the eligibility trace decay (also referred to as λ, which, if equal to zero, indicates that only values of the current stage receive an update).

*Model-based.* MB agents extend the model-free algorithm at the first stage by taking into account the transition structure P linking the first and second stages:

$${Q}_{MB}\left({s}_{A},{a}_{j}\right)=P\left({s}_{B} | {s}_{A},{a}_{j}\right)\underset{a\in \left\{{a}_{A},{a}_{B}\right\}}{\text{max}}{Q}_{MF}\left({s}_{B},a\right)+P\left({s}_{C} | {s}_{A},{a}_{j}\right)\underset{a\in \left\{{a}_{A},{a}_{B}\right\}}{\text{max}}{Q}_{MF}\left({s}_{C},a\right).$$

At the second stage, model-free and model-based agents perform equivalent updates, such that *Q*_*MF*_ = *Q*_*MB*_.

Hybrid. Hybrid agents arbitrate between the Q values according to a weighting parameter *w*:

$${Q}_{net}\left({s}_{A},{a}_{j}\right)=w{Q}_{MB}\left({s}_{A},{a}_{j}\right)+\left(1-w\right){Q}_{MF}\left({s}_{A},{a}_{j}\right).$$

*Decision rule.* Finally, Q values were subjected to a softmax function to determine choice probabilities:

$$P\left( {a_{i,t} = a|s_{i,t} } \right) = \frac{{e^{{\beta \left( {Q_{net} \left( {s_{i,t} ,a} \right) + \pi *rep\left( a \right) + \rho *resp\left( a \right)} \right)}} }}{{\sum {e^{{\beta \left( {Q_{net} \left( {s_{i,t} ,a^{\prime}} \right) + \pi *rep\left( {a^{\prime}} \right) + \rho *resp\left( {a^{\prime}} \right)} \right)}} } }}.$$

Here, β indicates the stochasticity of behavior, π a choice stickiness pareter (multiplied by rep(a) = 1 if first-stage action a was chosen on the current as well as the previous trial, otherwise zero), and ρ a response stickiness parameter (multiplied by resp(a) = 1 if the first-stage action a involved the same response key on the current as well as the previous trial, otherwise zero). We compared MF, MB and hybrid models excluding π and ρ (pure model), including π (+ choice stickiness model), and including π and ρ (+ choice + response stickiness model; see Supplement for more detail on parameter estimation and model fitting). The hybrid model including choice stickiness proved to have the most parsimonious fit, indicated by the Bayesian Information Criterion, and was used to retrieve individual behavioral task parameters, e.g., the weighting parameter *w*.

#### EEG recording and data reduction

EEG was recorded with Ag/AgCl electrodes from 61 sites of an equidistant electrode montage (Easycap GmbH, Breitbrunn, Germany) as well as from three external positions: approximately 2 cm below each eye to record eye movements and at the lower back to record the electrocardiogram. The EEG was amplified with two 32-channel BrainAmp amplifiers (Brain Products GmbH, Munich, Germany), recorded at a sampling rate of 500 Hz, and referenced to an electrode next to FCz. Offline, continuous data was filtered (0.1–30 Hz). After submitting data to an adaptive mixture independent component analysis (AMICA), we employed visual inspection together with the ICLabel toolbox^82^ to remove components containing eye-movement and cardioballistic artifacts. EEG data was then re-referenced to an average reference. Epochs of − 200 to 1000 ms around second-stage outcome presentation were subjected to adaptive artifact rejection, removing epochs containing deviations > 4 SDs of the mean probability distribution with the constraint to remove at least one and a maximum of 5% of trials or to otherwise adapt the SD threshold in steps of 0.1^83^. Epochs underwent a baseline correction (− 200 to 0 ms prior to outcome presentation) and trials including preliminary responses (reaction time < 100 ms) in either stage one or two were removed.

#### EEG analyses

##### First-level analyses

Electrophysiological data was subjected to single-trial analyses to quantify the relationship between EEG activity and trial-wise characteristics within our computational model. Second-stage feedback-locked data were used to investigate transition type and RPE. RPE was signed, i.e. could be positive or negative, combining valence and magnitude. We regressed EEG activity at each electrode and time point on trial type (common or rare) and RPE, using robust regression (EEG ~ Transition + RPE + Transition*RPE). The resulting temporo-spatial maps of *b* values per subject were then averaged over subjects to investigate whether transition type and RPE significantly accounted for variance in EEG activity. As we specifically assumed the FRN and P3 to reflect transition and RPE effects, analyses focused on the electrodes and time-windows corresponding to these event-related potentials (ERP). Search locations and intervals for each component were based on visual inspection of the raw EEG (grand averaged over all subjects). The FRN was thus determined as the negative EEG peak at FCz in a window of 250–350 ms after stimulus onset (peak latency: 294 ms) and the P3 as the positive peak between 330–430 ms at Cz (peak latency: 370 ms). *B* values were subjected to two-tailed one-sample t-tests against zero, employing false discovery rate^84^ (FDR) to correct for multiple comparisons.

##### Second-level analyses

To examine how first-level RPE signals varied as a function of impulsivity and compulsivity as well as the weighting of MF and MB control *(w)*, we examined their effect on first-level *b* values for the RPE effect. Since our first-level analyses revealed significant interaction effects of RPE and transition type at FCz, and Cz, suggesting that RPE is processed differently in common and rare trials, we conducted our further analyses separately within each transition type. In order to isolate the influence of impulsivity, compulsivity and *w* on distinct, RPE-related processes, analyses focused on the ERPs of interest (see above). We computed the mean first-level effect of RPE per subject by averaging the RPE *b* values in windows of − 25 ms and + 25 ms around the FRN- and P3-related peaks at FCz and Cz, respectively. These average RPE effects then served as dependent variables in robust linear regression models with z-scored impulsivity, compulsivity and *w* (weighting parameter) as well as their interactions as simultaneous predictors (mean *b* values ~ BIS-11 + OCI-R + *w* + BIS-11*OCI-R + BIS-11*w + OCI-R*w + BIS-11*OCI-R*w). Significant BIS-11*w interactions were followed up by regression analyses within high and low impulsivity and *w* score groups (based on median split). Pairs of regression coefficients were then compared in *z*-tests. Focusing on single values for the effect of RPE on the FRN and P3 allowed us to explore different regressors and their interactions without losing power due to correcting for multiple tests.

## Supplementary Information

Supplementary Information.

## Data Availability

Data and analysis routines are accessible under https://osf.io/vytdr/.
