# Supplementary material for: On the effects of impulsivity and compulsivity on neural correlates of model-based performance
Source: Sci Rep. 2024 Sep 10;14:21057. doi: 10.1038/s41598-024-71692-w (PMC11387645; doi:10.1038/s41598-024-71692-w)
Supplement: Supplementary file 1 — Supplementary Information. [file 41598_2024_71692_MOESM1_ESM.pdf]

Supplementary material for On the Effects of Impulsivity and Compulsivity on Neural Correlates of Model-based Performance

Kerstin Dück[1], Raoul Wüllhorst[1], Rebecca Overmeyer[1], Tanja Endrass[1,2]

[1] Faculty of Psychology, Chair for Addicition Research, Technische Universität Dresden, 01062 Dresden, Germany
[2] Neuroimaging Center, Technische Universität Dresden, 01062 Dresden, Germany

Computational modelling

Model fitting

We performed parameter estimation and model fit with the *fmincon* algorithm implemented in the *mfit* toolbox for MATLAB. This minimized the negative log likelihood across trials T, such that the probability of the model's choices given a set of parameters, $P(c_t \mid \theta)$ and those of the participant converged:

$$-LL = \sum_{t=1}^{T} \log P(c_t|\theta)$$

We used Bayesian Information Criterion (BIC) for model comparison, choosing the model with the lowest BIC mean, i.e. the most parsimonious fit. Starting values for the learning rate α, eligibility trace decay λ, and weighting w were drawn from flat distributions with 0 and 1 as lower and upper bounds. Inverse temperature β received starting values from gamma distribution with shape parameter = 4.82 and scale parameter = 0.88 as well as 0 and 20 as lower and upper bounds. Choice and response stickiness parameters π and ρ were drawn from normal distributions with $M \pm SD$ = 0.15±1.42 and -20 and 20 as lower and upper bounds.

Fitting results

We compared the fit (mean BIC) of models with different configurations of MF, MB and hybrid learners with additional model parameters (see Table S1). As expected, the hybrid models showed the best fit to the behavioral data[1]. The pure hybrid model (mean BIC = 983.05) was outperformed by a model including choice stickiness (mean BIC = 929.35). However, adding response stickiness (mean BIC = 929.38) did not improve the model. The hybrid + choice stickiness model also outperformed the MF and MB models with and without choice and response stickiness and was declared the winning model.

We obtained an inverse temperature of *M*±*SD* = 4.88±1.37, a learning rate of *M*±*SD* = 0.82±0.18, an eligibility trace decay of *M*±*SD* = 0.58±0.39, and a choice stickiness of *M*±*SD* = 0.19±0.09 from this winning model.

Table S1. Model fitting results

Model		mean BIC
hybrid	Pure	983.05
	+ choice stickiness	**929.35**
	+ choice + response stickiness	929.38
MB	Pure	998.36
	+ choice stickiness	931.45
	+ choice + response stickiness	931.50
MF	Pure	1057.70
	+ choice stickiness	978.11
	+ choice + response stickiness	977.85

Notes. Hybrid = hybrid agent arbitrating between model-based and model-free control according to the weighting parameter *w*. MB = model-based agent. MF = model-free agent. Pure = model without any additional parameters. BIC = Bayesian Information Criterion.

Reaction time difference as a marker for MB learning

Table S2. Robust regression on reaction time difference (rare minus common)

	β	*SE*	*t*	*p*
(Intercept)	351.63	9.39	37.44	<.001
BIS	-8.30	9.49	-.88	.380
OCI	1.28	9.41	.14	.892
BIS*OCI	-7.52	9.28	-.82	.410

Notes. BIS = z-scored sum score 11th Barratt Impulsiveness Scale. OCI = z-scored sum score Obsessive Compulsive Inventory Revised

Prolonged second-stage reaction times (RT) after rare transitions, indicating surprise, are based on knowledge on the transition structure, i.e., a mental model of the task[2]. RT difference was thus analyzed as a marker for MB learning. Regression analysis revealed neither main, nor interaction effects with impulsivity or compulsivity.

Post-hoc tests for BIS-11*w interaction

Table S3. Results of robust linear regressions for the FRN and P3 predicted by BIS-11 or *w* scores in median split groups of the other predictor

				predictor *w*			predictor BIS-11		
			(Intercept)	β	*SE*	z_Δ	β	*SE*	z_Δ
FRN common	BIS	high	2.48	-.03	.19	2.03*			
		low	1.79	.49	.16				
	w	high	2.63				.17	.22	.86
		low	1.66				.40	.15	
P3 rare	BIS	high	-.10	.34	.23	1.22			
		low	.31	-.09	.26				
	w	high	.18				.22	.27	1.83
		low	.04				-.43	.23	

Notes. Predictor *w* = models with *w* score as predictor. Predictor BIS-11 = models with BIS-11 score as predictor. FRN common = regressions of RPE effect in the time-window for feedback-related negativity at FCz after common transitions. P3 rare = regressions of RPE effect in the time-window for P3 at Cz after rare transitions. BIS = z-scored sum score 11th Barratt Impulsiveness Scale. W = z-scored weighting parameter. High = high median split group. Low = low median split group. z_Δ = z-test for difference between regression coefficients.
* Values $|z| > 1.96$ were considered significant ($\alpha = .95$).

First-level regression for transition*RPE interaction

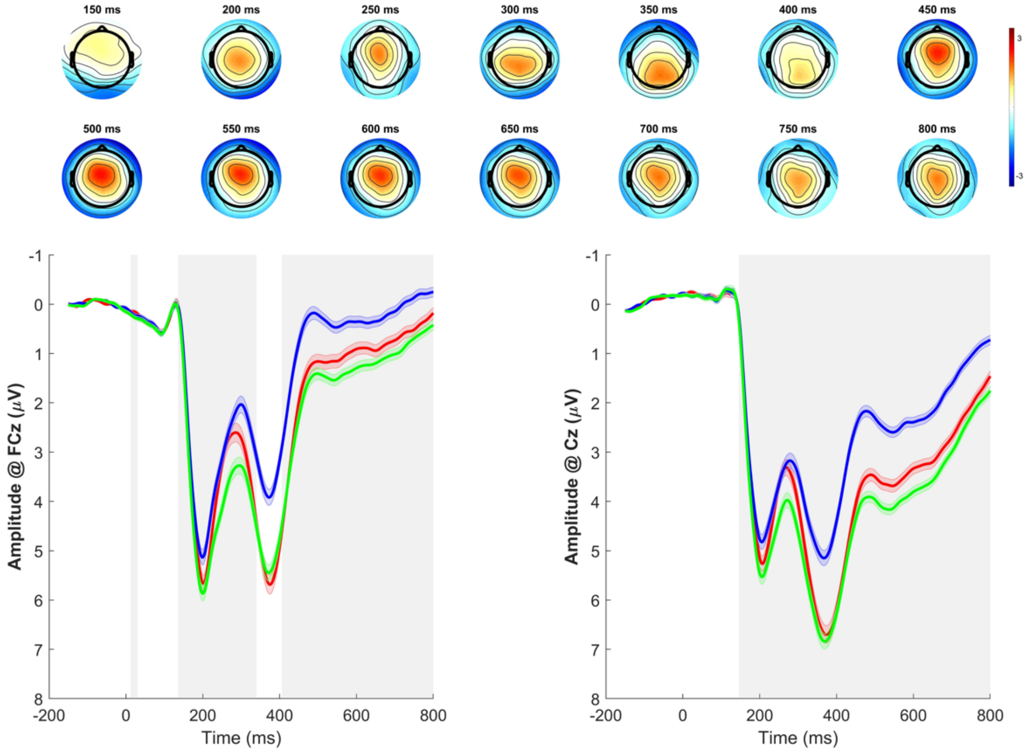

Figure S1. First-level regression for transition*RPE interaction at FCz (left) and Cz (right). First and second row: Topography of the *b* values for the Transition*RPE interaction (150-800 ms). Third row: EEG time course at FCz (left) and Cz (right). Shading indicates SEM. EEG activity is locked to second-stage feedback presentation. Gray shading behind EEG activity indicates significance of regression weights after FDR-correction.

References

1 Kool W, Cushman FA, Gershman SJ. When Does Model-Based Control Pay Off? *Plos Comput Biol* 2016; **12**: e1005090.

2 Seow TXF, Benoit E, Dempsey C, Jennings M, Maxwell A, O'Connell R *et al.* Model-Based Planning Deficits in Compulsivity Are Linked to Faulty Neural Representations of Task Structure. *J Neurosci* 2021; **41**: 6539–6550.
